# Long noncoding RNA CCAT1 acts as an oncogene and promotes chemoresistance in docetaxel-resistant lung adenocarcinoma cells

**DOI:** 10.18632/oncotarget.11518

**Published:** 2016-08-23

**Authors:** Jing Chen, Kai Zhang, Haizhu Song, Rui Wang, Xiaoyuan Chu, Longbang Chen

**Affiliations:** ^1^ Department of Medical Oncology, Jinling Hospital, School of Medicine, Nanjing University, Nanjing, Jiangsu, China

**Keywords:** lung adenocarcinoma (LAD), lncRNA CCAT1, let-7c, chemoresistance, epithelial-to-mesenchymal transition

## Abstract

Chemoresistance remains one of the major obstacles in clinical treatment of lung adenocarcinoma (LAD). Indeed, docetaxel-resistant LAD cells present chemoresistance and epithelial-to-mesenchymal transition phenotypes. Long non-coding RNAs (lncRNAs) are known to promote tumorigenesis in many cancer types. Here, we showed that the lncRNA colon cancer-associated transcript-1 (CCAT1) was upregulated in docetaxel-resistant LAD cells. Furthermore, downregulation of CCAT1 decreased chemoresistance, inhibited proliferation, enhanced apoptosis and reversed the epithelial-to-mesenchymal transition phenotype of docetaxel-resistant LAD cells. We also found that the oncogenic function of CCAT1 in docetaxel-resistant LAD cells depended on the sponging of let-7c. In turn, the sponging of let-7c by CCAT1 released Bcl-xl (a let-7c target), thereby promoting the acquisition of chemoresistance and epithelial-to-mesenchymal transition phenotypes in docetaxel-resistant LAD cells. Our data reveal a novel pathway underlying chemoresistance and the epithelial-to-mesenchymal transition in docetaxel-resistant LAD cells.

## INTRODUCTION

Lung cancer has among the highest morbidity and mortality rates of all malignancies worldwide. Approximately 70–80% of lung cancers are nonsmall-cell lung cancer, among which lung adenocarcinoma (LAD) is the most common type [1]. Although progress in clinical and experimental oncology has been made in recent years, the prognosis of LAD patients is still dismal [2]. Chemotherapy is a significant component of current first-line treatment for LAD patients. Docetaxel, a semisynthetic analogue of paclitaxel, is widely used in the treatment of LAD with genotoxic effects attributed to the induction of apoptosis and cell cycle arrest [3]. However, chemoresistance remains a major impediment to clinical application of this drug. Additionally, chemotherapy-induced epithelial-to-mesenchymal transition (EMT) in tumor cells is associated with chemoresistance [4–6]. Therefore, understanding the mechanisms underlying chemoresistance and chemotherapy-induced EMT in LAD might uncover new molecular targets that could be exploited for therapeutic benefits.

Long noncoding RNAs (lncRNAs; > 200 nucleotides) play significant roles in tumorigenesis at various levels, including chromatin modification, transcription, and post-transcriptional processing [7–11]. Recently, a novel level of post-transcriptional regulation has been proposed in which lncRNAs interact with miRNAs, functioning as competing endogenous RNAs (ceRNAs) [12–14].

Colon-cancer-associated transcript-1 (CCAT1), a 2,628-bp lncRNA located on chromosome 8q24.21, is abnormally expressed in colon cancer and promotes tumor progression [15]. CCAT1 is upregulated in hepatocellular carcinoma (HCC), gastric carcinoma, gallbladder cancer (GBC), and colon carcinoma tissues compared with adjacent normal tissues [16–19]. However, the expression of CCAT1 and its functional mechanisms in docetaxel-resistant LAD are still unclear.

In the current study, we comprehensively investigated the function of CCAT1 in docetaxel-resistant LAD cells. Our results showed that CCAT1 was upregulated in LAD tissues and promoted the acquisition of chemoresistance and EMT phenotypes in docetaxel-resistant LAD by competitively sponging up let-7c.

## RESULTS

### CCAT1 is upregulated in human LAD tissues and docetaxel-resistant cell lines

**Figure 1:**
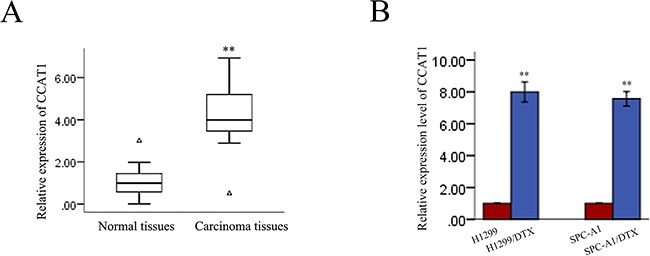
CCAT1 expression in LAD and its association with patient prognosis **A.** Differences in CCAT1 expression levels between LAD tissues and pair-matched noncancerous tissues. The expression of CCAT1 was normalized to that of GAPDH. Statistical differences between samples were analyzed with paired samples t-test (n = 36, p<0.01). **B.** Expression level of CCAT1 in parental and docetaxel-resistant LAD cell lines. Data are presented as mean ± standard error based on at least three independent experiments. *p<0.05, **p<0.01.

Chemoresistance has been a major obstacle in the clinical treatment of LAD. To determine whether CCAT1 is involved in the molecular etiology of chemoresistance in LAD, we first examined the expression of CCAT1 in 36 pairs of LAD tissues and pair-matched histologically normal tissues by quantitative reverse-transcription polymerase chain reaction (qRT-PCR). Compared with matched noncancerous tissue (Figure 1A), CCAT1 levels were increased in cancerous tissues (p<0.01). Next, we examined the expression of CCAT1 in two docetaxel-resistant LAD cell lines (SPC-A1/DTX and H1299/DTX) and the corresponding parental cell lines (SPC-A1 and H1299). As presented in Figure 1B, increased expression of CCAT1 was observed in docetaxel-resistant LAD cell lines compared with the parental cell lines. These results indicated that CCAT1 might be involved in the generation of the chemoresistance phenotype of LAD.

### Expression of CCAT1 influences cell proliferation, apoptosis, and docetaxel sensitivity of LAD *in vitro*

**Figure 2:**
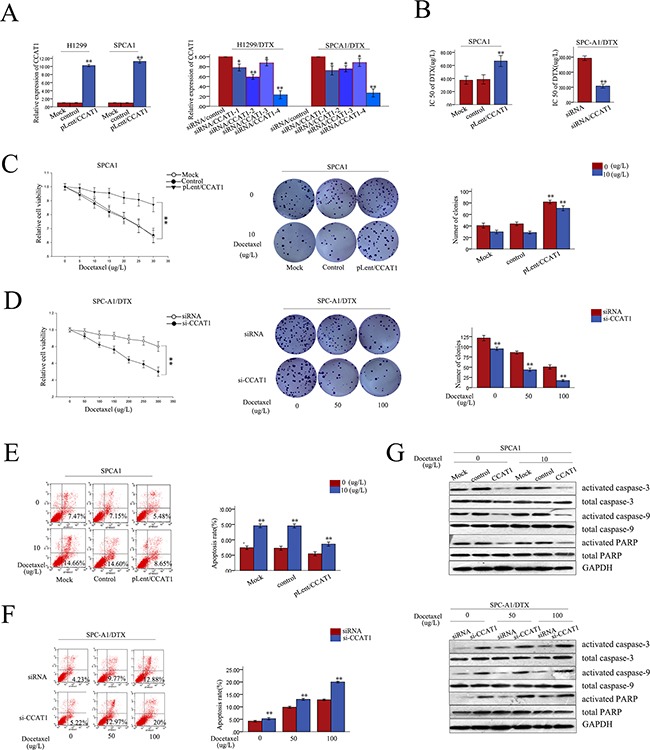
Role of CCAT1 in chemosensitivity of parental or docetaxel-resistant LAD cells **A.** qRT-PCR assay was performed to examine the expression of CCAT1 48 h after transfection of SPC-A1 or H1299 cells with CCAT1 (or control) and of SPC-A1/DTX or H1299/DTX cells with si-CCAT1 (or siRNA control). Cells transfected with null were regarded as Mock. **B.** IC50 values for docetaxel in SPC-A1 cells transfected with CCAT1 and SPC-A1/DTX cells transfected with si-CCAT1. **C.-D.** MTT and colony formation assays on SPC-A1 cells transfected with CCAT1, and SPC-A1/DTX cells transfected with siCCAT1. **E.-F.** Flow cytometry of SPC-A1 cells transfected with CCAT1, and SPC-A1/DTX cells transfected with siCCAT1. **G.** Western blot of apoptosis related proteins (activated caspase-3, total caspas-3, activated caspase-9, total caspase-9, activated PARP and total PARP). Error bars represent the mean ± SEM of at least three independent experiments. *p<0.05, **p<0.01 vs. control group.

To investigate the biological functions of CCAT1 in chemoresistance of LAD against docetaxel, SPC-A1 (or H1299) and SPC-A1/DTX (or H1299/DTX), cells were stably transfected with CCAT1 expression vector pLent/CCAT1 or CCAT1-specific siRNA, respectively, using the empty vector or control siRNA as a negative control (NC). Satisfactory transfection efficiency was obtained at 48 h post-transfection (Figure 2A). First, we examined the difference between the different concentration of docetaxel on the overexpression and the downregulation of CCAT1 by qRT-PCR. The overexpression and the downregulation of CCAT1 were unaltered when the cells were exposed to different concentration of docetaxel (Supplementary Figure S1E). We also measured the IC50 value for docetaxel in response to CCAT1 overexpression and downregulation. Compared with SPC-A1/control (or H1299/control) cells, the IC50 value of docetaxel in SPC-A1/pLent/CCAT1 (or H1299/pLent/CCAT1) increased by 85.7% (or 62.3%) (p<0.01, Supplementary Figure S2B, Supplementary Figure S1E). Conversely, compared with SPC-A1/DTX (or H1299/DTX) cells transfected with si-control, the IC50 value of docetaxel in SPC-A1/DTX (or H1299/DTX) transfected with si-CCAT1 was reduced by 55.12% (or 64.3%), (p<0.01, p<0.01, Figure 2B, Supplementary Figure S1D). MTT assays revealed enhanced proliferation of SPC-A1(or H1299) cells transfected with CCAT1 compared with NC-transfected cells when exposed to different concentrations of docetaxel (p<0.01; Figure 2C, Supplementary Figure S1A). On the other hand, suppressed proliferation was observed in CCAT1-downregulated SPC-A1/DTX (or H1299/DTX) cells compared with NC-transfected cells exposed to different concentrations of docetaxel (p<0.01; Figure 2D, Supplementary Figure S1A). Additionally, colony formation assays revealed higher proliferation of SPC-A1 (or H1299) cells transfected with CCAT1 compared with NC-transfected cells when treated with docetaxel (0 μg/L or 10 μg/L), with higher docetaxel concentrations promoting proliferation more strongly (p<0.01; Figure 2C, Supplementary Figure S1B). In contrast, suppression of proliferation was observed in CCAT1-downregulated SPC-A1/DTX (or H1299/DTX) cells compared with NC-transfected cells exposed to different concentrations of docetaxel (0 μg/L, 50 μg/L, or 100 μg/L). On the other hand, proliferation was suppressed when CCAT1 was downregulated (p<0.01; Figure 2D, Supplementary Figure S1B). Furthermore, we used flow cytometry analyses to assess the effect of CCAT1 on apoptosis. Compared with negative controls, forced expression of CCAT1 caused a decrease in apoptosis in SPC-A1 (or H1299) cells treated with docetaxel (0 μg/L or 10 μg/L). With the increasing docetaxel concentrations, apoptosis inhibition by CCAT1 was more apparent (p<0.01, Figure 2E and Supplementary Figure S1C). Likewise, downregulation of CCAT1 resulted in increased apoptosis in SPC-A1/DTX (or H1299/DTX) cells compared with the NC group when exposed to docetaxel (0 μg/L, 50 μg/L, or 100 μg/L, p<0.01), and such apoptosis-promoting effect was greater with higher concentration of docetaxel (Figure 2F and Supplementary Figure S1C). Next, we measured the levels of apoptosis-related proteins (activated caspase-3, total caspase-3, activated caspase-9, total caspase-9, activated PARP and total PARP proteins). Overexpression of CCAT1 increased the levels of *activated* caspase-3, -9, and PRAP in SPC-A1 (or H1299) and in SPC-A1/DTX (or SPC-A1/DTX) cells while downregulation of CCAT1 decreased it; however, total proteins levels remained unchanged (Figure 2G and Supplementary Figure S2A)). Therefore, CCAT1 might activate the caspase-3-dependent apoptosis pathway. Taken together, these data suggest that CCAT1 overexpression decreased the *in vitro* chemosensitivity of LAD cell, while enhancing their proliferation and reducing apoptosis.

### Expression of CCAT1 was associated with acquisition of an EMT phenotype in docetaxel-resistant LAD cells

**Figure 3:**
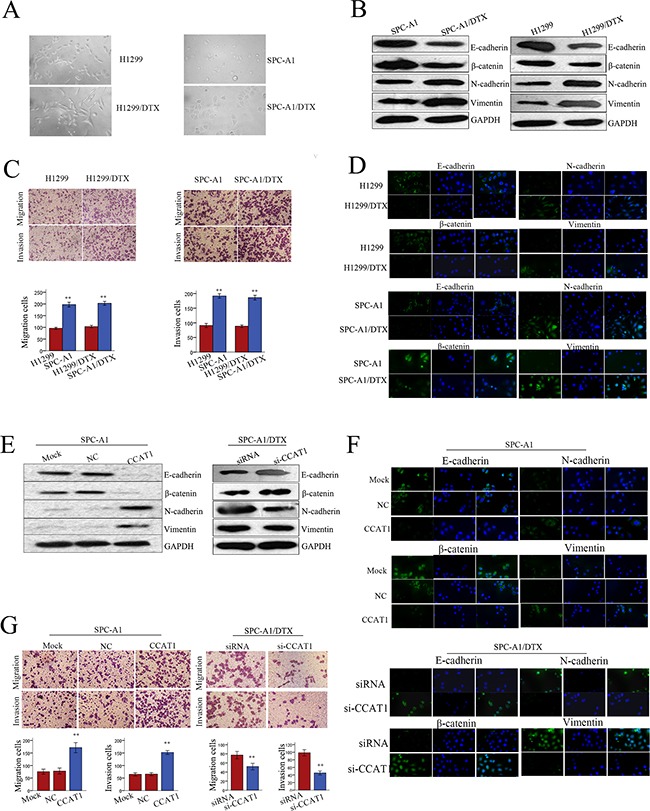
Forced expression of CCAT1 facilitates acquisition of an EMT phenotype in LAD cells **A.** The phenotype of docetaxel-resistant LAD cells and the corresponding parental cells. The docetaxel-resistant LAD cells present a fibroblast-like morphology (typical of mesenchymal phenotype) while the corresponding parental cells present a round-like morphology (typical of epithelial phenotype). **B.** Western blot of epithelial markers (E-cadherin, β-catenin) and mesenchymal markers (N-cadherin, vimentin), **C.** transwell assay to measure metastasis capacity, and **D.** immunofluorescence analysis of EMT markers in docetaxel-resistant LAD cells and parental LAD cells. **E.** Western blot and **F.** Immunofluorescence analysis of epithelial markers (E-cadherin, β-catenin) and mesenchymal markers (N-cadherin, vimentin), and **G.** transwell assay to measure metastasis capacity in SPC-A1 cells transfected with CCAT1, and in SPC-A1/DTX cells transfected with si-CCAT1. Error bars represent the mean ± SEM of at least three independent experiments. *p<0.05, **p<0.01 vs. control group.

EMT, a biological process in which cancer cells lose their epithelial polarity and undergo transition into a mesenchymal phenotype, plays a key role in cancer cell malignant transformation. Docetaxel-resistant LAD cells present a fibroblast-like morphology, which is typical of the mesenchymal phenotype of cells associated with the loss of epithelial markers compared with the corresponding parental cells (Figure 3A). Although there have been some studies on the contribution of the EMT phenotype in docetaxel-resistant LAD cells, much less is known about the role of CCAT1 during EMT. Therefore, we investigated whether the EMT phenotype of docetaxel-resistant LAD cells was affected by CCAT1 expression.

Western blotting and immunofluorescence were performed to test whether the EMT phenotype existed in docetaxel-resistant LAD cells. The expression of epithelial markers (E-cadherin, β-catenin) was decreased, while expression of mesenchymal markers (N-cadherin, vimentin), which are positively correlated with EMT, was increased in SPC-A1/DTX or H1299/DTX cells compared with parental cells (Figure 3B and 3D). Additionally, cell migration/invasion assays further confirmed the metastatic ability of LAD cells, as presented in Fig. 3C. To analyze the relationship between CCAT1 and the formation of the EMT phenotype in docetaxel-resistant LAD cells, we measured the levels of epithelial and mesenchymal markers in SPC-A1 (or H1299) and SPC-A1/DTX (or H1299/DTX) cells in response to different levels of CCAT1. As shown in Figure 3E and Figure S2A, forced expression of CCAT1 reduced the expression of epithelial markers and increased the expression of mesenchymal markers. Conversely, downregulation of CCAT1 increased the levels of epithelial markers and decreased the levels of mesenchymal markers. Moreover, results obtained from immunofluorescence studies showed a similar change in marker expression (Figure 3F and Supplementary Figure S2B). Cell migration/invasion assays revealed a facilitating effect of CCAT1 on metastasis of parental LAD cells. In contrast, SPC-A1/DTX (or H1299/DTX) cells transfected with si-CCAT1 showed relatively low migration and invasion capability compared with negative control groups (Figure 3G and Supplementary Figure S2C). Consequently, CCAT1 could be an important regulator of the EMT phenotype in docetaxel-resistant LAD cells.

### Effect of CCAT1 on chemoresistance and EMT of docetaxel-resistant LAD cells *in vivo*

**Figure 4:**
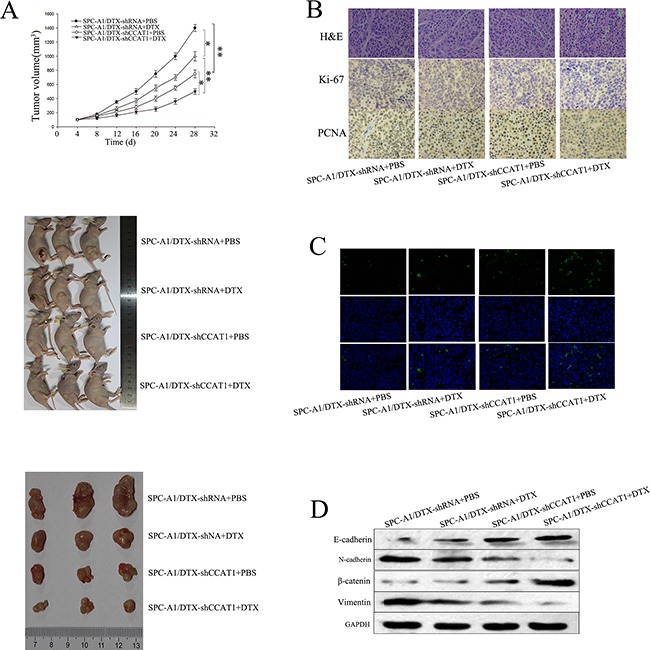
Effect of CCAT1 on chemoresistance and EMT of docetaxel-resistant LAD cells *in vivo* SPC-A1/DTX cells were transfected with shRNA-CCAT1 or shRNA-control and injected subcutaneously into nude mice. When the average tumor size reached approximately 50 mm^3^, docetaxel was administered through intraperitoneal injection at a dose of 1 mg/kg every other day for three doses in total. **A.** Growth curve of tumor volumes and representative photographs of tumor-bearing mice and tumors formed 28 days after the first administration of docetaxel. **B.-C.** Immunostaining of Ki-67, and PCNA proteins and TUNEL-stained sections of the transplanted tumors (original magnification, ×400). **D.** Western blot of epithelial markers (E-cadherin, β-catenin) and mesenchymal markers (N-cadherin, vimentin). *p<0.05, **p<0.01 vs. control group.

To assess the effects of CCAT1 on chemosensitivity and chemotherapy-induced EMT in docetaxel-resistant LAD cells *in vivo*, we inoculated nude mice with SPC-A1/DTX cells stably transfected with CCAT1 shRNA. Tumors derived from sh-CCAT1 transfected SPC-A1/DTX cells grew more slowly than those derived from control shRNA transfected cells after treatment with docetaxel (Figure 4A). Immunostaining analysis and TUNEL staining revealed a lower positive rate of proliferating cell nuclear antigen (PCNA) and Ki67, as well as a higher apoptotic rate in tumors derived from sh-CCAT1 transfected docetaxel-resistant LAD cells compared with the control groups (Figures 4B and 4C). Furthermore, we performed western blotting to detect the expression of epithelial and mesenchymal markers in subcutaneous tumors formed from cells transfected with sh-CCAT1. As shown in Figure 4D, the levels of epithelial protein markers were increased, while those of mesenchymal markers were decreased, compared with SPC-A1/DTX /shRNA-control. Together, these data suggest that dysregulated CCAT1 might be associated with the phenotypes of chemoresistance and chemotherapy-induced EMT in docetaxel-resistant LAD cells.

### Identification of Let-7c as a target of CCAT1

**Figure 5:**
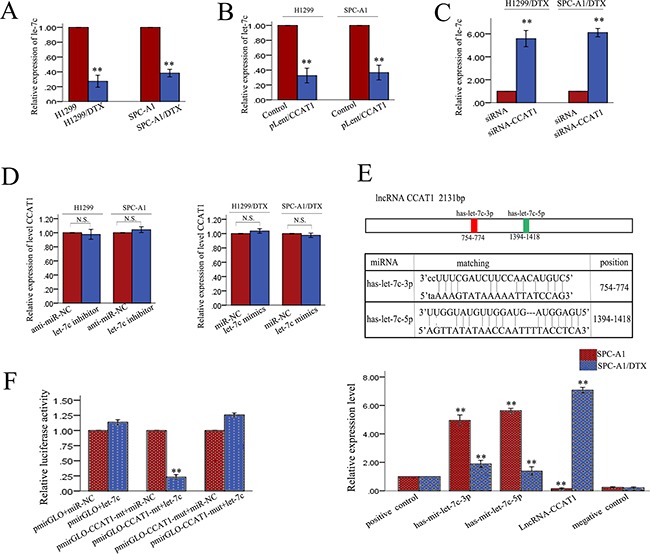
Let-7c is a target of CCAT1 **A.** qRT-PCR analysis of let-7c in parental and docetaxel-resistant LAD cells. **B.-C.** Effect of dysregulated CCAT1 expression on the level of let-7c measured by qRT-PCR. **D.** CCAT1 levels measured by qRT-PCR after ectopic expression or knockdown of let-7c. **E.** Top: Bioinformatics predicted let-7c binding sites at two distinct positions in CCAT1. Bottom: RIP assay to detect the association between CCAT1 and let-7c in SPC-A1 and SPC-A1/DTX cells. The positive and negative control refer to U1 and IgG, respectively. **F.** Luciferase activity in SPC-A1 cells co-transfected with let-7 and luciferase reporters containing no insert, CCAT1, or mutant CCAT1 (containing two mutations, let-7c-3p and let-7c-5p). Data are presented as the relative ratio of firefly luciferase activity to Renilla luciferase activity. N.S. means “not significant”, *p<0.05, **p<0.01 vs. control group.

Many lncRNAs are known to function as ceRNAs for specific miRNAs. CCAT1 was shown to physically associate with let-7 and act as a sponge for let-7. We performed a search for miRNAs that have complementary base pairing with lncRNA CCAT1, using the online software program starbase v2.0 (http://starbase.sysu.edu.cn/mirLncRNA.php), along with considering previously reported miRNAs. The search results demonstrated that 26 miRNAs formed complementary base pairing with CCAT1 (Supplementary Table S1). We measured the expression levels of these 26 miRNAs in CCAT1 downregulated LAD cells compared with control cells. We found four miRNAs that were upregulated more than 3-fold in CCAT1 downregulated LAD cells compared with control cells. We then focused on let-7c, which exhibited the greatest change. Since our previous study showed that let-7c expression enhanced the sensitivity of docetaxel-resistant LAD cells to chemotherapeutic agents and reversed their EMT phenotype, we hypothesized that the functions of CCAT1 in docetaxel-resistant LAD cells might be mediated by let-7c. To test this hypothesis, we first examined the expression levels of let-7c in SPC-A1/DTX and H1299/DTX cells and corresponding parental cells. As illustrated in Figure 5A, the expression levels of let-7c in SPC-A1/DTX and cells H1299/DTX was lower than those in the corresponding parental cells. To determine the relationship between CCAT1 and let-7c, we examined the expression level of let-7 in response to dysregulated CCAT1 expression. As shown in Figure 5B and 5C, the expression level of let-7c correlated negatively with that of CCAT1. Additionally, there was no difference in CCAT1 levels after ectopic expression or knockdown of let-7c (Figure 5D). To further validate the regulatory relationship between CCAT1 and let-7c, we performed an RNA immunoprecipitation (RIP) assay that revealed a competitive relationship between CCAT1 and let-7c (Figure 5E). The results of luciferase reporter assays provided further confirmation. As shown in Figure 5F, let-7c mimics reduced the luciferase activity of wild-type (WT) CCAT1 reporter vector but not that of empty vector and mutant reporter vector. These data confirmed the direct binding between CCAT1 and let-7c.

### Let-7c negatively regulates chemosensitivity, proliferation, apoptosis, and EMT phenotype of LAD cells *in vitro*

**Figure 6:**
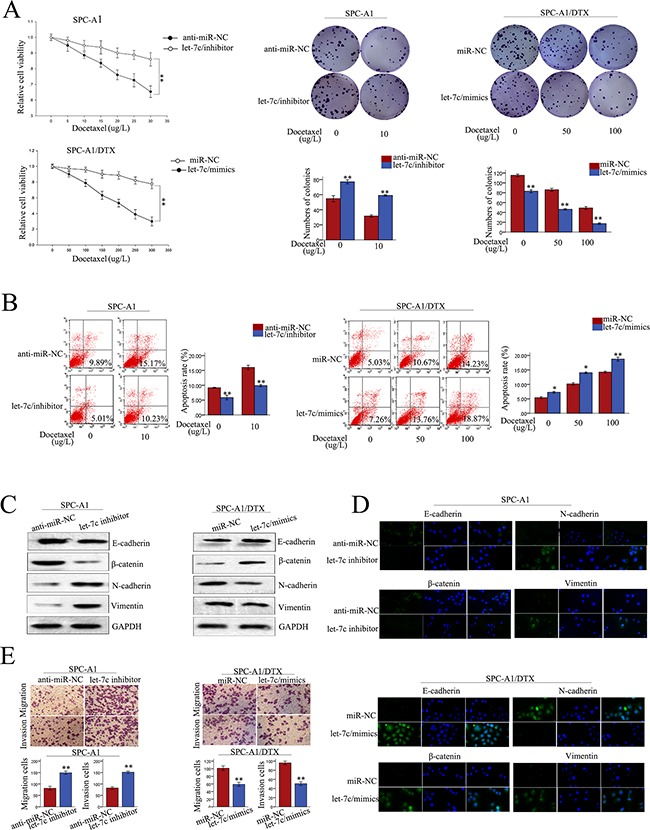
Role of let-7c in chemoresistance of docetaxel-resistant LAD cells **A.** MTT and colony formation assays, **B.** flow cytometry, **C.** western blot of epithelial markers (E-cadherin, β-catenin) and mesenchymal markers (N-cadherin, vimentin), **D.** immunofluorescence analysis of changes in epithelial and mesenchymal markers, and **E.** transwell assay to test for metastasis capacity of let-7c inhibitor-transfected SPC-A1 cells and let-7c mimics-transfected SPC-A1/DTX cells. Error bars represent the mean ± SEM of at least three independent experiments. *p<0.05, **p<0.01 vs. control group.

To investigate the biological function of let-7c in LAD cells, miR-NC or let-7c mimics were transfected into SPC-A1/DTX (or H1299/DTX cells) and anti-miR-NC or let-7c inhibitor was transfected into SPC-A1 (or H1299) cells. Satisfactory transfection efficiency was obtained after 48 h (Supplementary Figure S1D). The IC50 value for docetaxel in SPC-A1 (or H1299) cells transfected with let-7c inhibitor was increased by 71.79% (or 63.84%), respectively. On the other hand, the IC50 value for docetaxel in SPC-A1/DTX (or H1299/DTX) cells transfected with let-7c mimics, was decreased by 57.55% (or 60.56%), respectively, compared with the NC (p<0.01) (Supplementary Figure S1E). MTT and colony formation assays revealed enhanced proliferation of SPC-A1 (or H1299) cells transfected with let-7c inhibitor, and the opposite effect for SPC-A1/DTX (or H1299/DTX) transfected with let-7c mimics (p<0.01; Figure 6A, Supplementary Figure S1A and S1B). Moreover, flow cytometry analyses of apoptosis showed that let-7c inhibitor dramatically decreased the apoptosis of SPC-A1 (or H1299) cells, and the opposite result was obtained for SPC-A1/DTX (or H1299/DTX) cells transfected with let-7c mimics (p<0.01; Figure 6B, Supplementary Figure S1C). Additionally, western blot analysis was performed to investigate the function of let-7c on the induction of EMT in LAD cells. The expression of epithelial markers was decreased while that of mesenchymal markers was increased following transfection of let-7c inhibitor into SPC-A1 (or H1299 cells). The opposite results were obtained for SPC-A1/DTX (or H1299/DTX) transfected with let-7c mimic (Figure 6C, Supplementary Figure S2A). Results obtained from immunofluorescence studies further suggested that let-7c promotes the formation of EMT in LAD cells (Figure 6D, Supplementary Figure S2B). Moreover, transwell assays revealed increased migration and invasion of SPC-A1 (or H1299) cells in the presence of let-7c inhibitor. In contrast, the opposite results were obtained for SPC-A1/DTX (or H1299/DTX) cells treated with let-7c mimics (Figure 6E, Supplementary Figure S2C).

The above results suggest that let-7c negatively might regulate docetaxel chemosensitivity, proliferation, apoptosis, and EMT of LAD cells, thereby promoting the oncogenic function of CCAT1 in LAD.

### The oncogenic function of CCAT1 in LAD cells *in vitro* was dependent on let-7c

**Figure 7:**
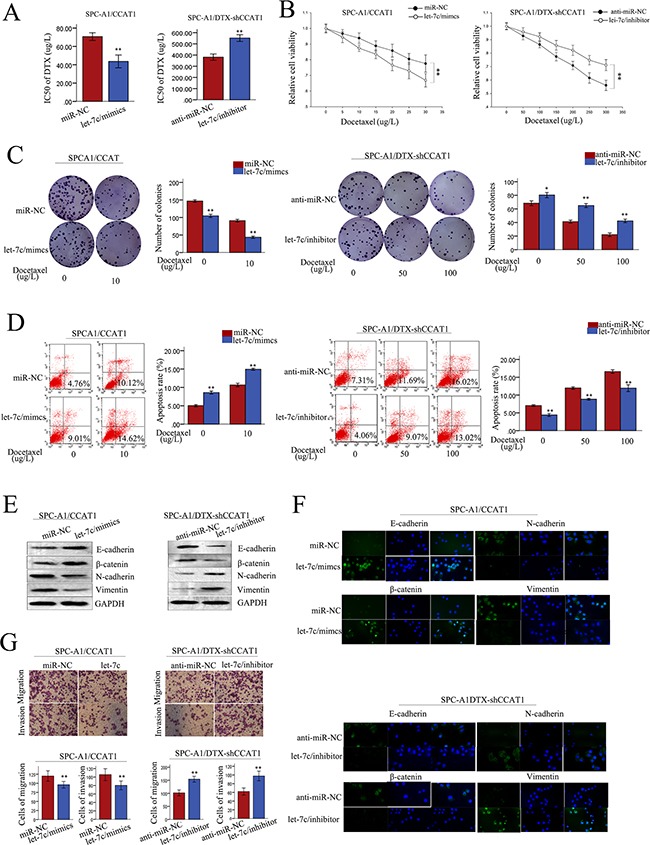
The function of CCAT1 in chemoresistance of LAD cells was partially reversed by let-7c LAD cells were stably transfected with pLent/CCAT1 or control vector and docetaxel-resistant LAD cells were stably transfected with sh-CCAT1 or shRNA-control. **A.** IC50 values of SPC-A1 cells stably transfected with pLent/CCAT1 and SPC-A1/DTX cells stably transfected with sh-CCAT1 was partially reversed by let-7c mimics and let-7c inhibitor, respectively. **B.-C.** MTT and colony formation assays were performed to determine the proliferation capacity of SPC-A1 cells stably transfected with pLent/CCAT1 and SPC-A1/DTX cells stably transfected with sh-CCAT1 in the presence of let-7c mimics and let-7c inhibitor, respectively. **D.** The apoptosis rate of SPC-A1 cells stably transfected with pLent/CCAT1 and SPC-A1/DTX cells stably transfected with sh-CCAT1 was partially reversed by let-7c mimics and let-7c inhibitor, respectively. Error bars represent the mean ± SEM of at least three independent experiments. *p<0.05, **p<0.01 vs. control group. **E.** The expression of epithelial markers (E-cadherin, β-catenin) and mesenchymal markers in SPC-A1 cells stably transfected with pLent/CCAT1 and SPC-A1/DTX cells stably transfected with sh-CCAT1 detected by western blotting was partially reversed by let-7c mimics and let-7c inhibitor, respectively. **F.** Immunofluorescence analysis of changes in epithelial markers and mesenchymal markers in SPC-A1 cells stably transfected with pLent/CCAT1 and SPC-A1/DTX cells stably transfected with sh-CCAT1 after treatment with let-7c mimic or let-7c inhibitor, respectively. **G.** The metastasis capacity of SPC-A1 cells stably transfected with pLent/CCAT1 and SPC-A1/DTX cells stably transfected with sh-CCAT1 was partially reversed by let-7c mimics and let-7c inhibitor, respectively. Error bars represent the mean ± SEM of at least three independent experiments. *p<0.05, **p<0.01 vs. control group.

We performed rescue experiments to determine whether CCAT1 influenced LAD cell proliferation, apoptosis, and the induction of EMT in a let-7c-dependent manner. Anti-miR-NC or let-7c inhibitor were transfected into SPC-A1/DTX (or H1299/DTX) cells stably transfected with shRNA-control or sh-CCAT1, and miR-NC or let-7c mimics were transfected into SPC-A1 (or H1299) cells stably transfected with pLent/CCAT1. Increased IC50 values for docetaxel induced by CCAT1 in SPC-A1(or H1299) cells were partially abolished by co-transfection of let-7c mimics, and vice versa in the transfected SPC-A1/DTX (or H1299/DTX) cells (p<0.01; Figure 7A, Supplementary Figure S1E). MTT assays showed that the enhanced proliferation induced by CCAT1 in SPC-A1 (or H1299) cells was in part abrogated by the introduction of let-7c mimic, and vice versa in the transfected SPC-A1/DTX (or H1299/DTX) cells (p<0.01; Figure 7B, Supplementary Figure S1A). Likewise, the increased colony formation induced by CCAT1 in SPC-A1 (or H1299) cells exposed to docetaxel (0 μg/L or 10 μg/L) was abrogated by the introduction of let-7c mimics, and vice versa in SPC-A1/DTX (or H1299/DTX) cells treated with docetaxel (0 μg/L, 50 μg/L, or 100 μg/L, p<0.01; Figure 7C, Supplementary Figure S1B). Flow cytometry assays revealed that the antiapoptotic effect of CCAT1 could be partially reversed by the introduction of let-7c mimics into SPC-A1 (or H1299) cells treated with docetaxel, and a similar antiapoptotic effect after exposure to docetaxel (0 μg/L, 50 μg, or 100 μg/L) was also observed after co-transfection of sh-CCAT1 and let-7c inhibitors into docetaxel-resistant LAD cells (p<0.01; Figure 7D, Supplementary Figure S1C). Additionally, western blotting assays indicated that the EMT effect could be partially abrogated by let-7c mimics in SPC-A1 (or H1299) cells stably transfected with CCAT1, and the opposite results were observed in SPC-A1/DTX (or H1299/DTX) cells with stably suppressed CCAT1 when treated with let-7c inhibitor (p<0.01; Figure 7E, Supplementary Figure S2A). Immunofluorescence analysis showed that the high expression of mesenchymal markers and the low expression of epithelial markers induced by CCAT1 in SPC-A1 (or H1299) cells was partially reversed by let-7c mimics, and vice versa in SPC-A1/DTX (or H1299/DTX) cells (p<0.01; Figure 7F, Supplementary Figure S2B). Furthermore, the pro-metastasis effect of CCAT1 in SPC-A1 (or H1299) cells could be partly abolished by co-transfection with let-7c mimics, whereas the anti-metastasis effect induced by sh-CCAT1 in SPC-A1/DTX (or H1299/DTX) cells could be partially reversed by let-7c inhibitors (p<0.01; Figure 7G, Supplementary Figure S2C). These results showed that CCAT1 influences cell proliferation, apoptosis, docetaxel chemosensitivity, and the induction of the EMT phenotype in LAD cells *in vitro,* at least in part in a let-7c-dependent manner.

### CCAT1 positively regulates the let-7c target gene Bcl-xl in LAD tissues

**Figure 8:**
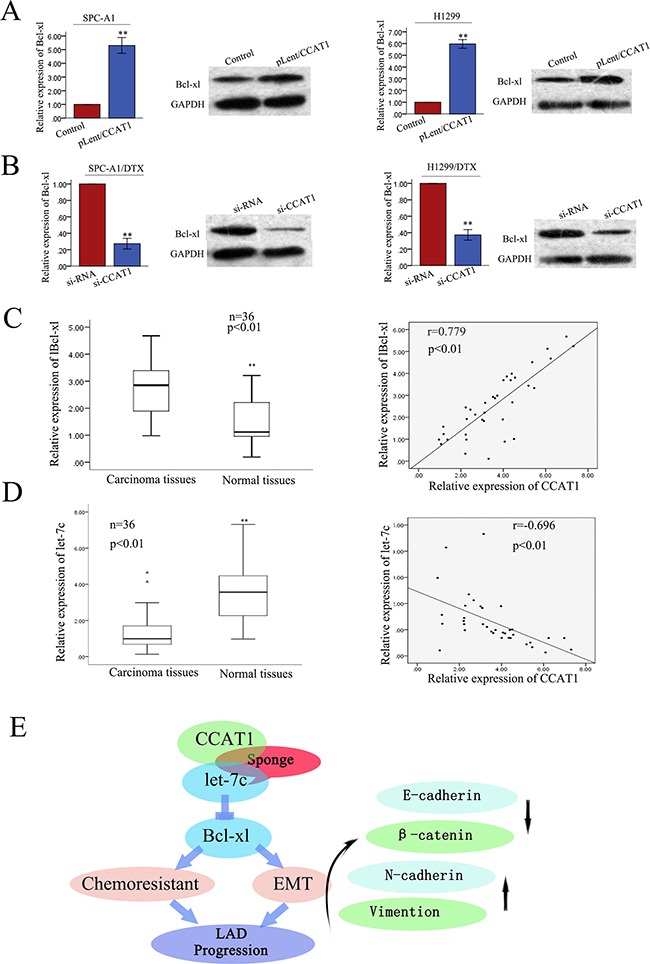
CCAT1 positively regulates the let-7c target gene Bcl-xl in LAD tissues **A.** The ectopic expression of CCAT1 upregulated Bcl-xl at both transcript and protein levels in two parental LAD cell lines. **B.** In docetaxel-resistant cells, si-CCAT1 decreased Bcl-xl at both transcript and protein levels. **C.** The expression of Bcl-xl in 36 paired samples of primary LAD and NT cells (p<0.01), showing positive correlation between Bcl-xl expression and CCAT1 expression in 36 LAD tissues (2-tailed Spearman's correlation, r=0.779, p<0.01). **D.** qRT-PCR detection of let-7c expression in 36 paired LAD and NT samples (p<0.01), showing negative correlation between CCAT1 and let-7c expression levels in 36 LAD tissues (Spearman's correlation analysis, r = −0.696; p<0.01). **E.** Schematic overview of CCAT1 regulatory signaling. Data are presented as mean ± SD of at least three independent experiments.

In our previously studies, we showed that let-7c can contribute to the acquisition of chemoresistance and EMT phenotypes by directly targeting Bcl-xl in docetaxel-resistant LAD cell. We proposed that CCAT1 and Bcl-xl interact with let-7c by functioning as ceRNAs. Here, to confirm such model, we measured the level of Bcl-xl in response to different levels of CCAT1. We observed that in SPC-A1 and H1299 cells the ectopic expression of CCAT1 upregulated Bcl-xl at the transcript and protein levels (Figure 8A). In SPC-A1/DTX and H1299/DTX cells, si-CCAT1 decreased Bcl-xl at the transcript and protein levels (Figure 8B). Furthermore, the expression of Bcl-xl in 36 paired samples of primary LAD and corresponding non-carcinoma tissues were determined by qRT-PCR. The relative levels of Bcl-xl in LAD tissues were significantly higher than those in the corresponding normal tissues (p<0.01, Figure 8C) Additionally, Bcl-xl levels and CCAT1 levels were positively correlated (2-tailed Spearman's correlation, r = 0.779, p<0.01; Figure 8C). Furthermore, the expression of let-7c in 36 paired samples of primary LAD and corresponding non-carcinoma tissues was also measured by qRT-PCR, which showed that the levels of let-7c in LAD tissues were significantly lower than those in the corresponding normal tissues. Furthermore, let-7c levels and CCAT1 levels correlated negatively (2-tailed Spearman's correlation, r = −0.696, p<0.01; Figure 8D). Collectively, these findings indicated that there is a regulatory signaling pathway in which CCAT1 regulates Bcl-xl by competitively sponging up let-7c, inducing increased chemoresistance and an EMT phenotype in docetaxel-resistant LAD cells (Figure 8E).

## DISCUSSION

NcRNAs, including miRNAs, have been shown to promote tumorigenesis [20–26]. Recently, the dysregulation of lncRNAs has also been shown to contribute to cancer pathogenesis, providing novel therapeutic opportunities to treat cancer [27–29]. However, the roles of lncRNAs in LAD carcinogenesis are not well understood. Investigating the molecular mechanism by which lncRNAs function in LAD might facilitate the exploitation of novel therapeutic targets. Docetaxel-resistant LAD cells exhibit reduced sensitivity to docetaxel and acquire an EMT phenotype. Chemoresistance is one of the main obstacles in the clinical treatment of patients diagnosed with LAD. Moreover, the emergence of EMT contributes to the malignancy of LAD. Therefore, investigating the mechanisms underlying chemoresistance and the EMT phenotype in LAD might facilitate the development of novel treatments that improve patient prognosis. In the present study, we provide evidence that CCAT1 acts as an oncogene by sponging let-7c, highlighting CCAT1 as a potential prognostic marker and therapeutic target for LAD.

Endogenous transcripts containing miRNA response elements (MREs) can co-regulate each other by acting as miRNA sponges or ceRNAs, forming large-scale regulatory networks across the transcriptome [13, 30, 31]. Multiple studies have documented that lncRNAs harbor potential MREs and function as competitive platforms for miRNAs in multiple types of cancer [25, 32–35]. Such findings warrant investigating the lncRNA-miRNA interaction in LAD and other types of cancer.

CCAT1, located in the vicinity of c-MYC, is abnormally expressed in colon cancer and is dysregulated in many other cancers [17, 19, 36–41]. However, the mechanism by which CCAT1 exerts its oncogenic functions in the tumorigenesis of LAD remains unclear. Although chemotherapy is one of the main treatment methods for LAD, especially in patients diagnosed with advanced LAD, chemoresistance represents a major obstacle to clinical treatment of LAD. In this study, we first showed that CCAT1 was upregulated in LAD tissues compared with pair-matched noncancerous tissues. Additionally, we comprehensively investigated the functions of CCAT1 in docetaxel-resistant LAD cells by employing gain-of-function and loss-of-function approaches. Forced expression of CCAT1 facilitated the proliferation and migration of LAD cells, implicating that CCAT1 promoted the acquisition of chemoresistance and EMT phenotypes in docetaxel-resistance LAD.

Emerging evidence suggests that lncRNAs act as sponges for common miRNAs and abolish the endogenous suppressive effect of these miRNAs on their *bona fide* targets. CCAT1 may function as a part of the ceRNA network [42]. Ma *et al.* demonstrated that CCAT1 was upregulated in GBC tissues and that overexpression of CCAT1 facilitated the proliferation and invasiveness of GBC cells by competitively sponging miR-218-5p [19]. In addition, CCAT1 levels are increased in HCC tissues compared with pair-matched noncancerous hepatic tissues [37]. Forced expression of CCAT1 promoted HCC cell proliferation and migration, which was dependent upon competitive binding with let-7 [37]. The mature forms of the let-7 family members are highly conserved across species and encode an evolutionarily conserved family of 13 homologous miRNAs located in genomic regions that are frequently deleted in human cancer [43]. Dysregulation of let-7c suppresses proliferation in lung cancer and inhibits the metastatic capacity of colorectal cancer cells [44, 45]. In one of our previous studies, we discovered that let-7c expression increased the sensitivity of docetaxel-resistant LAD cells to chemotherapeutic agents and reversed their EMT phenotype [46]. Therefore, we hypothesized that CCAT1 might function in LAD in a let-7c–dependent manner. To test this hypothesis, we performed RIP and luciferase reporter assays to confirm the association between CCAT1 and let-7c. In addition, let-7c inhibitor yielded very similar effects to ectopic CCAT1 expression in LAD cells. Thus, we conclude that let-7c is a *bona fide* CCAT1-targeting miRNA and suggest that CCAT1 might function as a ceRNA for let-7c.

Silencing of CCAT1 increased the chemosensitivity of docetaxel-resistant LAD cells, whereas overexpression of CCAT1 decreased it. Furthermore, overexpression of CCAT1 contributed to chemotherapy-induced EMT. Our data suggest that the function of CCAT1 in LAD cells is partially exerted via competitive sponging of let-7c, preventing the inhibition of Bcl-xl. Our study provides new insight into the mechanisms underlying chemoresistance and chemotherapy-induced EMT of LAD by revealing a novel regulatory pathway, which may be targeted for therapeutic benefits.

## MATERIALS AND METHODS

### Patients

LAD tissues (n=36) and pair-matched noncancerous tissues were obtained from patients diagnosed with advanced LAD at the Department of Medical Oncology, Jinling Hospital (Nanjing, PR China) between March 2011 and September 2015. Informed consent was obtained from patients.

### Cell lines

Two human LAD cell lines, SPC-A1 and H1299, were purchased from the Tumor Cell Bank of the Chinese Academy of Medical Science (Shanghai, China) and cultured in RPMI 1640 medium containing 10% fetal bovine serum and ampicillin and streptomycin at 37°C in a humidified atmosphere of 95% air and 5% CO2. The docetaxel-resistant LAD cells (SPC-A1/DTX and H1299/DTX) derived from parental SPC-A1 and H1299 cells, respectively, were established and preserved in 50 μg/L final concentration of docetaxel.

### Real-time quantitative reverse-transcription polymerase chain reaction (qRT-PCR)

Total RNA from tissues and cells was isolated with Trizol reagent (Invitrogen, CA, USA) according to the manufacturer's instructions. Reverse transcription was performed with PrimeScript RT reagent Kit (Takara, Japan) according to the manufacturer's protocol. qRT-PCR was performed with SYBR Prime Script RT-PCR Kits (Takara, Japan) based on the manufacturer's instructions. The CCAT1 or let-7c level was calculated with the 2^ΔΔCt^ method, which were normalized to GAPDH mRNA or U6 rRNA, respectively. All assays were performed in triplicate. The expression levels were relative to the fold change of the corresponding controls, which were defined as 1.0. PCR primers were designed as follows: CCAT1 forward:

5′-TCATTACCAGCTGCCGTGTT-3′, and reverse: 5′-TCATGTCTCGGCACCTTTCC-3′; GAPDH: forward, 5′-CTGGGCTACACTGAGCACC-3′, and reverse: 5′- AAGTGGTCGTTGAGGGCAATG-3′; let-7c: forward: 5′-GGTTGAGGTAGTAGGTTGTATGGT-3′, and reverse: 5′-AACATGTACAGTCCATGGATG-3′; U6 forward: 5′-CGCTTCGGCAGCACATATACTA-3′, and reverse: 5′-CGCTTCACGAATTTGCGTGTCA-3′.

### Cell transfection

Hsa-miRNA-let-7c mimic/negative control mimic and hsa-miRNA-let-7c inhibitor/negative control inhibitor were purchased from Applied Biological Materials (ABM, Canada). The cDNA encoding CCAT1 was PCR-amplified and subcloned into the pLenti-GIII-CMV-Puro vector (ABM, Canada), which was named pLent/CCAT1. The siRNAs specifically targeting CCAT1 were synthesized by ABM (Canada). The siRNA sequences for CCAT1 were si-CCAT1-1, 5′-CCTGGCCCTCTCATCAGAGACTTGACTTA-3′, si-CCAT1-2, 5′-GATGTGTGAGTCCTAATTGAAATGAGGCC-3′, si-CCAT1-3, 5′AGGCAGAAAGCCGTATCTTAATTATTGCA-3′and si-CCAT1-4, 5′TGACTTGATCTTTGAACTTTAGCTCA CCA-3′. Transfections were performed using the Lipofectamine 2000 kit (Invitrogen) according to the manufacturer's instructions.

### Construction of stable cell lines with overexpression or downregulation of CCAT1

Cell lines stably expressing CCAT1, H1299 or SPC-A1 cells were transfected with the plasmid pLent/CCAT1, and screened with Puromycin (2μg/ml) for four weeks. Cell lines stably suppressing CCAT1 were constructed by transfection with a lentivirus construct containing the desired vector, and screened with Puromycin (2 μg/ml) for four weeks.

### Dual luciferase reporter assay

PmirGLO, pmirGLO-CCAT1wt or pmirGLOCCAT1-mut (let-7c) was co-transfected with let-7c mimics or miRNA NC into SPC-A1 cells by Lipofectamine-mediated gene transfer. The relative luciferase activity was normalized to Renilla luciferase activity 48 h after transfection. The data were relative to the fold change of the corresponding control groups defined as 1.0.

### *In vitro* chemosensitivity assay

Chemosensitivity was measured by 3-(4,5- dimethylthiazol-2-yl)-2,5-diphenyl-tetrazolium bromide (MTT, Sigma, USA) assay. Cells were cultured in 96-well plates treated with docetaxel. After 48 h, the MTT solution (5 mg/ml, 20 μl) was added to each well. Following incubation for 4 h, the media was removed and 100 μl DMSO were added to each well. The relative number of surviving cells was assessed by measuring the optical density (O.D.) of cell lysates at 560 nm. All assays were performed in triplicate.

### Cell viability

Cells were seeded into 96-well plates (3 × 10^3^ cells/well) directly or 24 h after transfection. After treatment with the indicated drug combinations for 48 h, cell viability was assessed via 3-(4,5-dimethylthiazol-2-yl)-2, 5-diphenyl-trtrazolium bromide (MTT) assay.

### Colony formation assay

Cells (500 cells/well) were plated in 6-well plates and incubated in RPMI 1640 with 10% FBS at 37°C. Two weeks later, the cells were fixed and stained with 0.1% crystal violet. The number of visible colonies was counted manually.

### Flow cytometry analysis to detect apoptosis

Cells transfected with the indicated plasmid or negative control were reaped after 48 h. Apoptosis was measured by performing flow cytometry analyses with Annexin V: FITC Apoptosis Detection Kits (BD Biosciences, USA), according to the manufacturer's instructions. All samples were assayed in triplicate.

### Cell migration and invasion assays

Cell migration and invasion were measured by transwell chamber (8 um pore size, Corning) and Matrigel invasion (Bection Dickinson), respectively. Forty-eight h after transfection, cells in serum-free media were placed into the upper chamber coated with or without 10 μg/ml Matrigel. Media containing 10% FBS was added into the lower chamber. Following 48 h incubation, cells remaining in upper membrane were wiped off, while cells that migrated or invaded were fixed in methanol, stained with 0.1% crystal violet and counted under a microscope. Three independent experiments were carried out.

### Immunofluorescence

Cells seeded on glass coverslips in 6-well plates were fixed in 4% formaldehyde solution and permeabilized with 0.5% Triton X-100/PBS. Cells were blocked with 5% BSA-PBS for 1 h at room temperature and incubated with primary antibody at 4°C overnight, followed by incubation with fluorescent-dye conjugated secondary antibody (Invitrogen) for 1 h, and then stained with DAPI. Finally, images were taken under an inverted fluorescence microscope.

### Western bolt analysis and antibodies

Total protein lysates were separated in 10% sodium dodecyl sulfate-polyacrylamide gel electrophoresis (SDS-PAGE), and were electrophoretically transferred to polyvinylidene difluoride membranes (Roche). Protein loading was estimated using mouse anti-GAPDH monoclonal antibody. The membranes were blotted with 10% non-fat milk in TBST for 2 h at room temperature, washed and then probed with the rabbit anti-human E-cadherin (1: 2000 dilution), β-catenin (1: 2000 dilution), N-cadherin (1: 2000 dilution), vimentin (1: 2000 dilution), activated caspase-3 (1: 2000 dilution), total caspase-3 (1: 2000 dilution), activated caspase-9 (1: 2000 dilution), total caspase-9 (1: 2000 dilution), activated PARP (1: 2000 dilution), total PARP (1: 2000 dilution), and GAPDH (1: 3000 dilution), overnight at 4°C, followed by treatment with secondary antibody conjugated to horseradish peroxidase for 2 h at room temperature. The proteins were detected using an enhanced chemiluminescence system and exposed to x-ray film. All antibodies were purchased from Abcam (USA).

### RNA immunoprecipitation (RIP)

RNA immunoprecipitation was performed using thermo fisher RIP kit (Thermo, USA) based on the manufacturer's protocol. The Ago2 antibodies were purchased from Abcam (USA). Normal mouse IgG (Abcam, USA) was applied as negative control and anti-SNRNP70 (Abcam, USA) was employed as positive control for the RIP procedure. Purified RNA was subjected to qRT-PCR analysis to demonstrate the presence of the binding targets using respective primers.

### Xenograft transplantation and immunohistochemistry

Approximately 5.0*10^6^ SPC-A1/DTX cells suspended in 100 μl PBS and stably transfected with shRNA/CCAT1 or shRNA/control were injected subcutaneously into the right side of the posterior flank of female BALB/c athymic nude mice (Department of Comparative Medicine, Jinling Hospital) at five to six weeks of age. Tumor growth was examined every other day with a vernier caliper. Tumor volumes were calculated by using the equation: V=A*B^2^/2 (mm^3^), where A is the largest diameter and B is the perpendicular diameter. When the average tumor size reached about 50 mm^3^, docetaxel was given through intraperitoneal injection with a concentration of 1.0 mg/kg, one dose every other day with three doses totally. After five weeks, all mice were killed and necropsies were carried out. The primary tumors were excised, paraffin-embedded, formalin-fixed, and conducted hematoxylin and eosin (H&E) staining, immunostaining analysis for Ki-67 and proliferating cell nuclear antigen (PCNA) protein expression according to the manufacturer's instructions.

### TUNEL assay

Apoptosis in transplanted-tumor tissues was detected using the TUNEL assay, performed according to the guidelines recommended by the TUNEL assay kit (KeyGen, Nanjing, China).

### Statistical analysis

Data are shown as the means ± standard error of at least three independent experiments. The SPSS 17.0 software (SPSS Inc., Chicago, IL, USA) was used for statistical analyses. Two group comparisons were performed with a Student t test. Multiple group comparisons were analyzed with one-way ANOVA. All tests performed were two-sided. Statistically significant negative correlation between CCAT1 and let-7c expression levels in LAD tissues from 36 cases was analyzed by Spearman's correlation analysis. Statistically significant positive correlation between CCAT1 and Bcl-xl expression levels in LAD tissues from 36 cases was analyzed by Spearman's correlation analysis. P < 0.05 was considered statistically significant.

## SUPPLEMENTARY FIGURES AND TABLE


