# Supplementary material for: Long noncoding RNA CCAT1 acts as an oncogene and promotes chemoresistance in docetaxel-resistant lung adenocarcinoma cells
Source: Oncotarget. 2016 Aug 23;7(38):62474–89. doi: 10.18632/oncotarget.11518 (PMC5308740; doi:10.18632/oncotarget.11518)
Supplement: Supplementary file 1 [file oncotarget-07-62474-s001.pdf]

Long noncoding RNA CCAT1 acts as an oncogene and promotes chemoresistance in docetaxel-resistant lung adenocarcinoma cells

SUPPLEMENTARY FIGURES AND TABLE

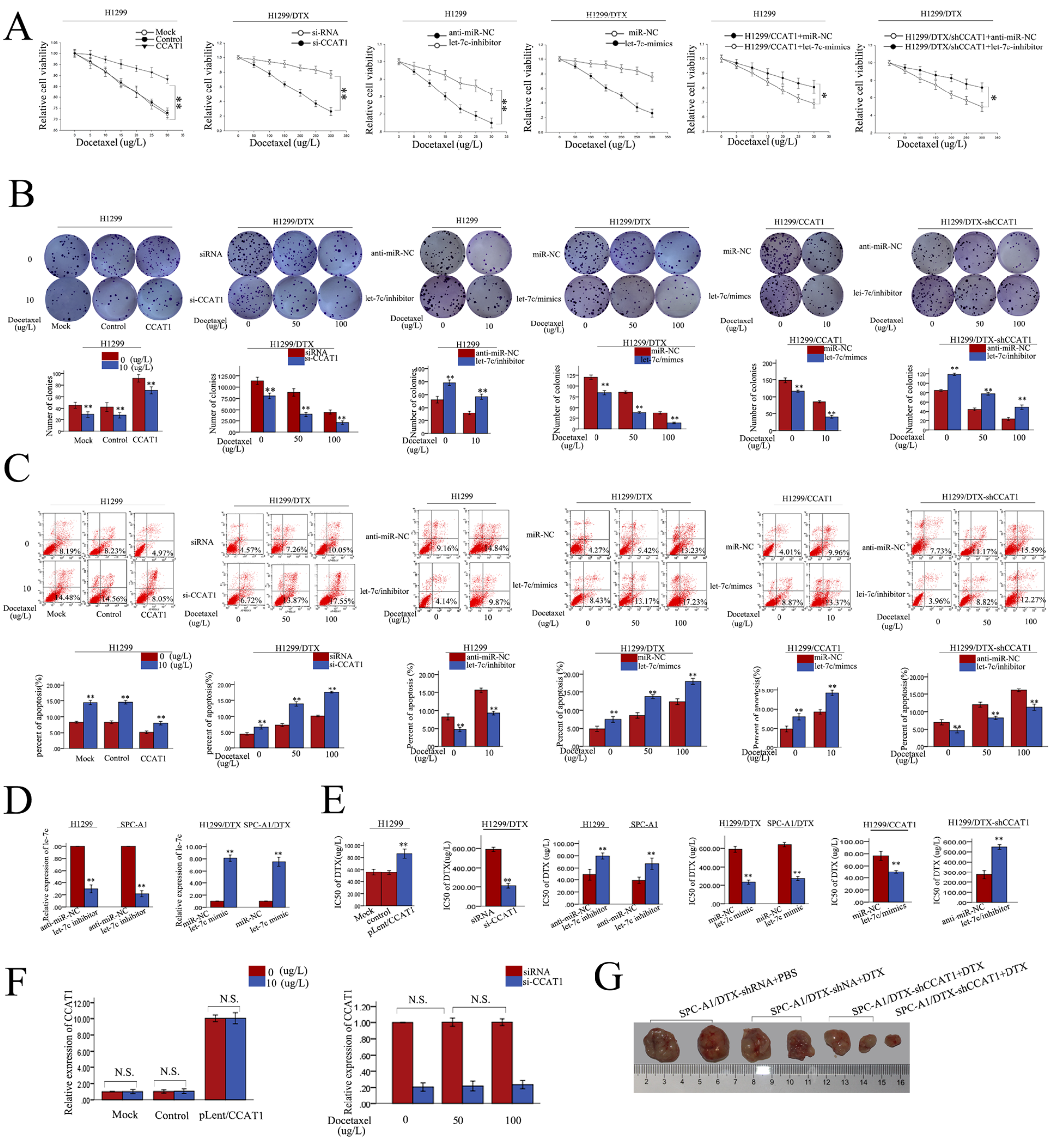

Supplementary Figure S1: A.MTT assays to measure cell viability, **B.** colony formation assays to measure cell proliferation, and **C.** flow cytometry analysis to measure apoptosis in (left to right) H1299 cells transfected with CCAT1, H1299/DTX cells transfected with si-CCAT1, H1299 cells transfected with let-7c inhibitor, H1299/DTX cells transfected with let-7c mimics, H1299/CCAT1 cells co-transfected with let-7c mimics, and H1299/DTX/shCCAT1 cells co-transfected with let-7c inhibitor. **D.** qRT-PCR was performed to measure transfection efficiency. SPC-A1 and H1299 cells were transfected with let-7c inhibitor while SPC-A1/DTX and H1299/DTX cells were transfected with let-7c mimics. **E.** MTT was performed to examine the IC50 of cells. Left to right: H1299 cells transfected with CCAT1, H1299/DTX cells transfected with si-CCAT1, H1299 and SPC-A1cells transfected with let-7c inhibitor, H1299/DTX and SPC-A1/DTX cells transfected with let-7c mimics, H1299/CCAT1 cells co-transfected with let-7c mimics, and H1299/DTX/shCCAT1 cells co-transfected with let-7c inhibitor. **F.** qRT-PCR was applied to compare the difference between the different concentration of docetaxel on the overexpression and the downregulation of CCAT1. **G.** Five mice were used in every group (N=5), and one mouse in the last group was dead.

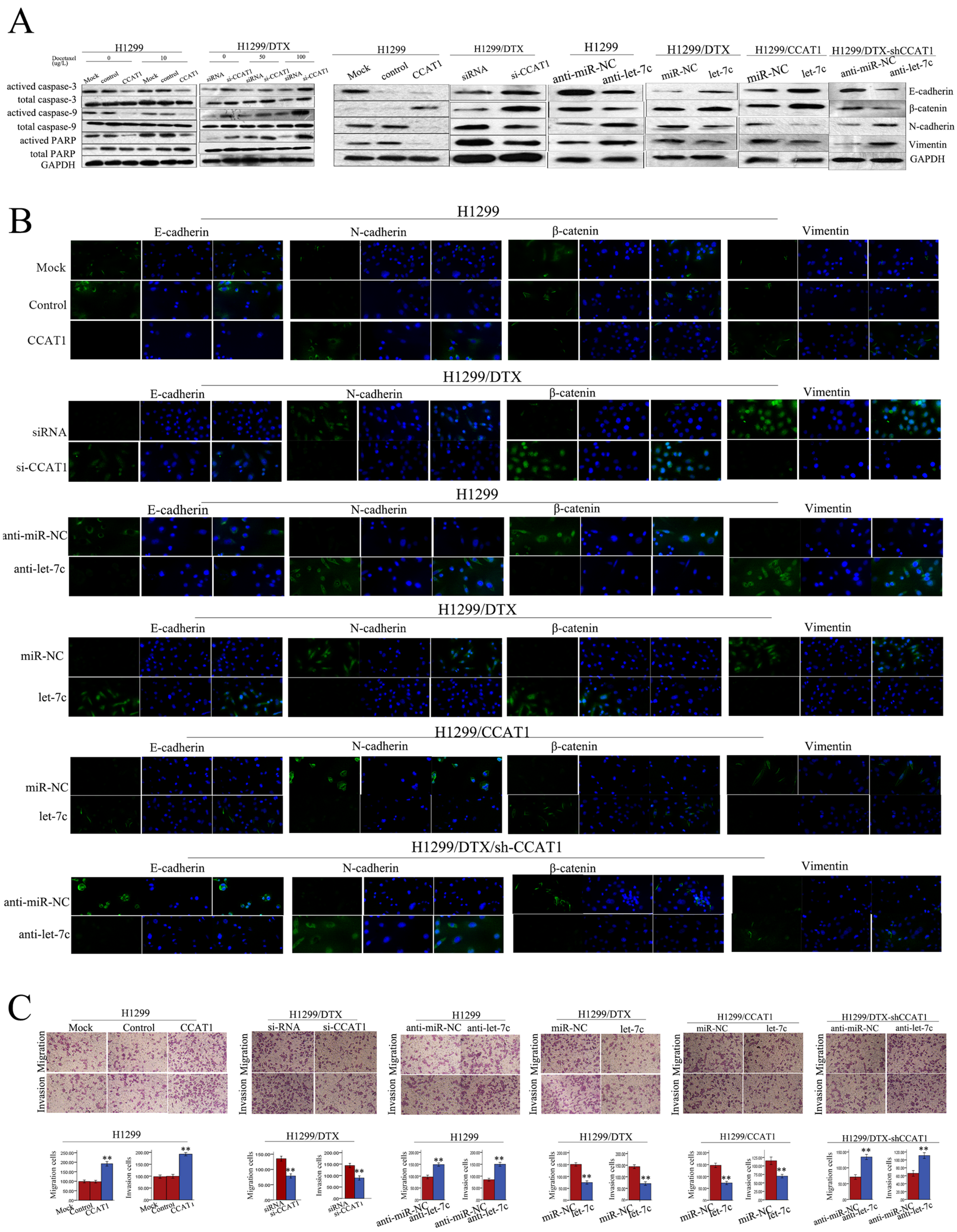

Supplementary Figure S2: A. Western blot and **B.** Immunofluorescence to detect EMT markers, and **C.** transwell assays to determine cell migration and invasion ability of (left to right) H1299 cells transfected with CCAT1, H1299/DTX cells transfected with si-CCAT1, H1299 cells transfected with let-7c inhibitor, H1299/DTX cells transfected with let-7c mimics, H1299/CCAT1 cells co-transfected with let-7c mimics, and H1299/DTX/shCCAT1 cells co-transfected with let-7c inhibitor.

Supplementary Table S1: Initial profiling of miRNAs in CCAT1 downregulated LAD cells (SPC-A1/DTX-shCCAT1) compared with the control cells (SPC-A1/DTX)

	miRNA	fold-change
1	has-miRNA-130a-3p	2.5172
2	has-miRNA-130b-3p	1.6315
3	has-miRNA-148a-3p	3.8173
4	has-miRNA-148b-3p	1.9506
5	has-miRNA-152-3p	0.2141
6	has-miRNA-181a-5p	0.4231
7	has-miRNA-181b-5p	0.6564
8	has-miRNA-181c-5p	0.4098
9	has-miRNA-181d-5p	0.4326
10	has-miRNA-216a-5p	0.1223
11	has-miRNA-218-5p	4.7044
12	has-miRNA-24-3p	0.3652
13	has-miRNA-296-3p	0.3697
14	has-miRNA-301a-3p	0.641
15	has-miRNA-301b	0.3306
16	has-miRNA-3666	0.6046
17	has-miRNA-410-3p	4.3524
18	has-miRNA-4295	0.6381
19	has-miRNA- 454-3p	2.5771
20	has-miRNA-4262	1.1676
21	has-miRNA-490-3p	0.3242
22	has-miRNA-543	1.3411
23	has-miRNA-let-7a	1.356
24	has-miRNA-let-7b	2.134
25	has-miRNA-let-7c	6.0174
26	has-miRNA-let-7e	1.0126
